# Twelve-month clinical outcomes of 206 patients with chronic pulmonary aspergillosis

**DOI:** 10.1371/journal.pone.0193732

**Published:** 2018-04-10

**Authors:** Felix Bongomin, Chris Harris, Gemma Hayes, Chris Kosmidis, David W. Denning

**Affiliations:** 1 The National Aspergillosis Centre, Wythenshawe Hospital, Manchester University NHS Foundation Trust, Manchester, United Kingdom; 2 Division of Infection, Immunity and Respiratory Medicine, School of Biological Sciences, Faculty of Biology, Medicine and Health, The University of Manchester, Manchester, United Kingdom; 3 The Manchester Academic Health Service Centre, Manchester, United Kingdom; Lee Kong Chian School of Medicine, SINGAPORE

## Abstract

There is a paucity of evidence surrounding the optimal antifungal therapy for use in chronic pulmonary aspergillosis (CPA) and the duration of therapy remains unclear. We retrospectively evaluated treatment outcomes, including change in quality of life scores (St George’s Respiratory Questionnaire (QoL)), weight and *Aspergillus* IgG at 6 and 12 months following initiation of therapy in a cohort of 206 CPA patients referred to the UK National Aspergillosis Centre (NAC), Manchester between April 2013 and March 2015. One hundred and forty-two patients (69%) were azole naïve at presentation and 105 (74%) (Group A) were commenced on itraconazole, 27 (19%) on voriconazole, and 10 (7%) were not treated medically. The remainder (64 patients, 31%) had previously trialled, or remained on, azole therapy at inclusion (Group B) of whom 46 (72%) received itraconazole, 16 (25%) voriconazole, and 2 (3%) posaconazole. Initial therapy was continued for 12 months in 78 patients (48%) of those treated; the azole was changed in 62 (32%) patients and discontinued in 56 (29%) patients for adverse reactions (32, 57%), azole resistance (11, 20%), clinical failure (8, 14%) or clinical stability (5, 9%). Azole discontinuation rates were higher in Group B than in Group A (42% vs. 22%, p = 0.003). For all patients who survived, weight increased (median of 62.2Kg at baseline, to 64.8 at 12 months), mean *Aspergillus* IgG declined from 260 (baseline) to 154 (12 months) and QoL improved from 62.2/100 (baseline) to 57.2/100 (12 months). At 12 months, there was no difference in median survival between Groups A and B (95% vs. 91%, p = 0.173). The rate of emergence of resistance during therapy was 13% for itraconazole compared to 5% for voriconazole. Bronchial artery embolization was done in 9 (4.4%) patients and lobectomy in 7 (3.2%). The optimal duration of azole therapy in CPA is undetermined due to the absence of evidenced based endpoints allowing clinical trials to be undertaken. However we have demonstrated itraconazole and voriconazole are modestly effective for CPA, especially if given for 12 months, but fewer than 50% of patients manage this duration. This suggests extended therapy may be required for demonstrable clinical improvement.

## Introduction

Chronic pulmonary aspergillosis (CPA) is an uncommon, slowly destructive pulmonary syndrome characterised by progressive cavitation, fibrosis, and pleural thickening. Most common in patients with underlying cavitating lung disease or structural pulmonary anomaly, CPA confers high morbidity and mortality [1]. The spectrum of CPA encompasses aspergilloma, *Aspergillus* nodules, chronic cavitary pulmonary aspergillosis (CCPA), sub-acute invasive aspergillosis (SAIA), and chronic fibrosing pulmonary aspergillosis (CFPA) [2]. Elements of CPA may co-exist, for example CCPA and aspergilloma [3].

The clinical course of CPA is highly variable between patients as is the associated pulmonary and systemic symptom burden. At present there is no established correlation between symptoms, associated biochemical and serological markers of infection, and disease severity [1,4,5]. Survival rates vary significantly among published series and may be affected by underlying pulmonary disease (58–93% at one year, 17.5%-85% at five years, 30–50% at ten years) [4,6–9].

The efficacy of treatment in CPA is not well established but anecdotally reduces symptom burden and may improve prognosis [10,11]. Current practice advocates long-term oral azole therapy, with frequent emergence of side effects or failure of therapy, leading to a change to a different azole or to an intravenous antifungal agent. Few studies assessing therapeutic efficacy exist and none provide a head to head comparison of available azole therapy. Most are small and of short duration. Patients not on azole therapy require careful follow up and early institution of therapy in the event of clinical, radiological or serological deterioration [12].

At the National Aspergillosis Centre, patients are usually initiated on itraconazole as primary therapy. Reasons for starting an alternative azole such as voriconazole include contraindications to itraconazole use, itraconazole resistance, and high burden of disease, such as presence of multiple aspergillomas. Regardless of the azole initiated, all patients undergo regular therapeutic drug monitoring and dose adjustment.

Establishing objective therapeutic response in CPA is difficult and at present there is no evidence to support the use of commonly used measures of therapeutic response, including *Aspergillus* IgG, weight, quality of life assessment questionnaires such as the St George’s Respiratory Questionnaire (SGRQ) and sputum microbiology [13,14]. Cross-sectional imaging appearances have recently been validated as useful markers of disease progression and remain the only evidence-based tool available for assessment of treatment response [15] Nonetheless a combination of clinical, radiological, microbiological and serological changes is commonly used to define response.

This retrospective single centre study aims to assess the ‘real life’ impact of oral triazole therapy on quality of life, body weight and serology over 12 months, specifically comparing itraconazole and voriconazole.

## Patients and methods

### Patients

**Fig 1:**
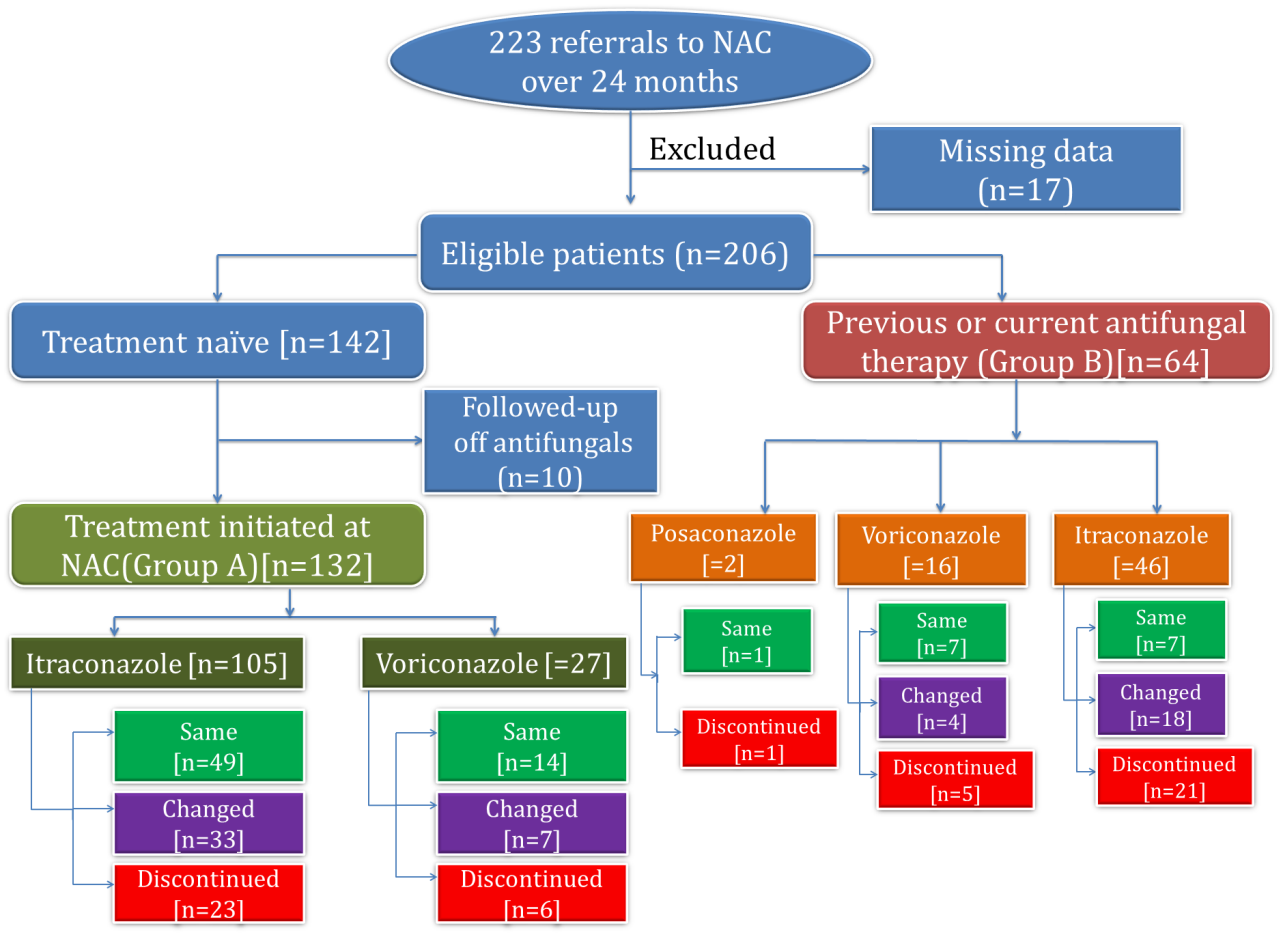
Patients selection, stratification and treatment course for all the patients referred to the National Aspergillosis Centre over 24 months. Abbreviation: **NAC**. National Aspergillosis Centre.

A retrospective review of 223 patients referred to the National Aspergillosis Centre (NAC), Manchester, UK over a 24-month period (April 2013 to March 2015) was performed. All patients were assessed against the recent European Society of Clinical Microbiology and Infectious Diseases and European Respiratory Society (ESCMID/ERS) guidelines to confirm the diagnosis of CPA [2]. Patients with other forms of aspergillosis were excluded. Data were collected at 0, 6 and 12 months following initial consultation and the clinical outcomes evaluated. Detailed evaluation was performed for those who met the CPA diagnostic criteria [2] and were commenced on treatment (Group A n = 132). Patients who were referred while on or previously treated with antifungal therapy for CPA were also evaluated (Group B n = 64). Ten treatment naïve patients were monitored off therapy. Patients (n = 17) with missing data were excluded from analysis (Fig 1).

### Data collection

Data collected at 0, 6 and 12 months included demographics (age, sex, and underlying pulmonary and systemic co-morbidities), *Aspergillus*-specific IgG titer (ImmunoCap (Phadia/ThermoFisher)), SGRQ scores, Medical Research Council (MRC) dyspnea score, weight and prior antifungal therapy. Alterations in azole therapy and the rationale for this were also documented. Survival rates were calculated at 12 months.

Date of azole initiation, dose, adverse drug events, and resistance during therapy, alteration or discontinuation of therapy and co-morbid conditions were retrieved from the patients’ case-notes. SGRQ scores, weight and MRC dyspnea scores at 0, 6 and 12 months were extracted from an established prospectively collated patient database established by the NAC. *Aspergillus*-specific IgG titers were extracted from electronic medical records. Radiological response to therapy, inflammatory markers, and therapeutic drug monitoring (TDM), culture results, and resistance mechanisms were not collected as part of the study.

### Definitions

***Same therapy***—refers to individuals who remained on the primary therapy documented at study inclusion for the entire study period, irrespective of dose or dosage adjustments.

***Changed therapy***—refers to cases where the primary therapy was changed to an alternative azole, and the subsequent therapy continued throughout the study period irrespective of dose or dosage adjustment.

***Discontinued therapy***—refers to cases where treatment was stopped within the study period and no replacement therapy commenced.

Being a retrospective study, patients’ informed consent and ethical review were waived. Data was collected as part of a clinical audit and was anonymized prior to analysis.

### Statistical analysis

Values are presented as frequencies (%) for qualitative variables and mean ± standard deviation (SD) for continuous, normally distributed variables or median and interquartile range for non-normally distributed variables. Survival was calculated from the date of initiation of the primary therapy (voriconazole or itraconazole) to the date of death or last follow-up (52 weeks) using the Kaplan–Meier (Mantel-Cox) test; a log-rank test was applied to compare survival curves. Statistical significance was taken as a p ≤0.05. Data analyses were performed with the use of IBM SPSS statistic software, version 23.0 (IBM Corp., Armonk, NY, USA) and GraphPad Prism version 7.00 for Windows (GraphPad Software, La Jolla California USA) was used for graphical illustrations.

## Results

### Demographic characteristics and underlying diseases

Of the 206 analysed, 10 (7%) treatment naïve patients were not initiated on any azole (Fig 1): two had aspergilloma resected, one had a stable simple aspergilloma, one was on ongoing tuberculosis (TB) therapy, and the rest (n = 6) had stable serology and radiological parameters and were being monitored off antifungals. One hundred and thirty two patients were referred to the NAC before commencement of antifungal therapy (Group A) (Fig 1). This cohort comprised of 84 males (64%) and 48 females (36%). The median age was 65 years (range: 34–86). Sixty-four (29%) patients were referred on or having previously been treated with azole therapy. These were analysed separately (Group B) (Fig 1). Forty–five (70%) of the 64 patients in Group B were males and the median age was 61 (range: 22–85) years. Both cohorts demonstrate a male bias consistent with the published literature.

Fourteen (7%) patients had no underlying pulmonary co-morbidity. The median number of underlying pulmonary comorbidities per patient was 2 (range 1–5). The overall frequency of underlying pulmonary diseases is summarised in Table 1. Almost 60% of the patients had COPD or previous mycobacterial infections. The frequency of underlying specific immune deficiency in CPA is unknown however it is worth noting that twenty patients (10%) had mannose binding lectin deficient within this cohort.

**Table 1:** Underlying and co-existing pulmonary disorders among patients with chronic pulmonary aspergillosis.

Underlying condition (overall)	Number	Percent
Chronic obstructive pulmonary disease	60	29
Tuberculosis	37	18
Non-tuberculous mycobacterial infections	20	10
Allergic bronchopulmonary aspergillosis	17	8
Bronchiectasis	10	5
Pneumothorax	9	4
Sarcoidosis	9	4
Lobectomy	8	4
Asthma	6	3
Previous lung cancer	5	2
Rheumatoid arthritis	4	2
Community acquired pneumonia	3	1
Ankylosing spondylitis	2	1
Asbestosis	1	0
Empyema thoracis	1	0
None	14	7
**Total**	**206**	**100**

### Initial and subsequent anti-fungal therapy

Of the 132 Group A patients, 105 (80%) were commenced on itraconazole and 27 (20%) on voriconazole (Fig 1). Overall, 63 (48%) patients in Group A remained on the primary therapy for 12 months, 29 (22%) had their therapy discontinued and 40 (30%) had their primary therapy replaced with an alternative antifungal agent.

Sixty-four patients were referred with prior or current antifungal therapy (Group B). The duration of prior antifungal therapy for these patients was often uncertain and difficult to ascertain due to patient recollection and commencement of treatment at a peripheral center. The majority of these individuals (72%, n = 46) had received itraconazole prior to referral although only 6 (13%) remained on it at enrollment. Voriconazole had been prescribed in 25% (n = 16) of individuals; nine (56%) were still on treatment when first seen. Two patients (3%) had previously received intravenous anti-fungal therapy (liposomal amphotericin B (n = 1) and micafungin (n = 1)). Posaconazole was given as initial therapy to two patients prior to referral.

### Itraconazole therapy

Forty-nine (47%) patients in Group A were on itraconazole therapy continuously over the 12 month period, 23 (22%) discontinued it and 33 (31%) were changed to a different azole. Twenty (61%) of the 33 patients whose regimen was changed were switched to voriconazole and 13 (39%) to posaconazole. The median duration of itraconazole therapy in Group A was 12 weeks (range 1–44) before changing to another triazole and 14 weeks (range: 1–48) before discontinuation of therapy. Only 2 (9%) patients had to stop itraconazole therapy before 12 weeks.

The median itraconazole dose administered in Group A patients was 400mg per day (range 200-400mg /day). Seventy (67%) patients were on 400mg/day, 19 (18%) on 300mg and 16 (15%) on 200mg per day. Doses were adjusted based on TDM and tolerance in 34 of the 49 (69%) patients who took itraconazole for 12 months. Fifteen (31%) patients remained on the same dose over the 12-month period. Twenty eight (57%) patients had their dose reduced due to persistently elevated therapeutic drug levels which placed them at greater risk of developing itraconazole driven side effects. The median reduction in itraconazole dose was 200mg/day (range 50-300mg lower per day). Six (12%) patients had their itraconazole dose increased by a median dose of 100mg (100-200mg) per day.

The majority of Group B patients were on an itraconazole dose of 400mg (100-600mg) daily (n = 34, 74%). Eighteen (39%) of the 46 patients who were referred while taking itraconazole changed therapy, 7 (15%) remained on the same therapy for 12 months and 21 (46%) had their therapy discontinued. Twelve patients (67%) were changed to voriconazole, five (28%) to posaconazole and one to isavuconazole. The reasons for a change of therapy were: 1) adverse events (n = 13, 72%), 2) lack of improvement (n = 4, 22%), and 3) resistance (n = 1, 6%). Fourteen (67%) of the 21 patients whose therapy had to be discontinued had adverse events, four (19%) had multi- or pan-azole resistant *A*. *fumigatus* and one patient was on anti-TB therapy for non-tuberculous mycobacteria (NTM).

Across both groups, 35 (33%) patients developed adverse events to itraconazole over the 12-month period, with 15 (43%) requiring discontinuation of therapy. Ten (5%) patients had clinical failure, 8 (8%) had itraconazole resistant *A*. *fumigatus* isolates (5 mono-resistance and 3 pan-azole resistance), and another three (13%) were clinically stable and were followed up off therapy. Adverse events leading to change or discontinuation of therapy are shown in Table 2.

**Table 2:** Adverse events leading to change or discontinuation of itraconazole therapy for both Group A and B patients.

Adverse events	Number	Percent
Ankle swelling	19	31
Gastro-intestinal disturbance	11	18
Peripheral neuropathy	10	16
Shortness of breath	5	8
Visual disturbance	4	6
Fatigue and weakness	3	5
Hepatoxicity	3	5
Alopecia	1	2
Cardiac toxicity	1	2
Severe headache	1	2
Photosensitivity	1	2
Confusion and memory loss	1	2
Loss of libido	1	2
Rhabdomyolysis	1	2
**Total**	**62**	**100**

### Voriconazole therapy

In Group A, the reasons for initiation of voriconazole included a contra-indication to itraconazole (notably heart failure), severe disease (including large aspergilloma), and isolation of *Aspergillus* spp. resistant to itraconazole but sensitive to voriconazole at the time of diagnosis. The median dose of voriconazole prescribed was 400mg/day (range 200-500mg/day). The median duration of voriconazole therapy in Group A was 14 weeks (range: 2–40) before change of regimen and 31 weeks (range: 15–46) before discontinuation. In no patient was voriconazole discontinued before 12 weeks of therapy.

Fourteen (52%) of the 27 patients in Group A were on voriconazole therapy for the whole observation period (12 months period). Therapy was switched for seven patients, 5 (71%) of whom were commenced on posaconazole and 2 (29%) on itraconazole. Voriconazole dose was reduced in half of the patients (n = 7) and the other half remained on the same dose for the whole study period. The median reduction in voriconazole dose was 100mg/day (range 100-250mg lower per day). No patient had his/her voriconazole dose increased.

In Group A, five of seven patients who changed therapy (71%) because of adverse events/intolerance (n = 3), in one (14%) to circumvent a drug interaction with therapy for concurrent *Mycobacterium avium* complex infection one because of persistently low levels of voriconazole on TDM. Voriconazole therapy was discontinued in 6 (21%) patients. One patient’s therapy was stopped because of resistance.

In Group B, the dose of voriconazole dose was at 400mg daily for 12 (75%) patients (range: 200-400mg). Of the 16 patients in Group B who were on voriconazole, 7 (44%) continued on the same therapy for the 12 months, treatment was discontinued for 5 (31%) patients, 4 (25%) patients changed therapy. Three (75%) of the patients changed to posaconazole and 1 (25%) changed to itraconazole. The reasons for change of therapy were: 1) adverse events (photosensitivity (n = 1) and visual disturbance (n = 1), 2) resistance (n = 1), and 3) lack of clinical improvement. Two (40%) of the 5 patients discontinued treatment due to voriconazole-related adverse events; three (60%) of the patients were clinically stable off therapy.

Across both groups, 10 (37%) patients developed adverse events, five (50%) of which were severe enough to need discontinuation of voriconazole (Table 3) and one (4%) patient discontinued voriconazole because of emergence of resistance.

**Table 3:** Adverse events leading to change or discontinuation of voriconazole therapy for both Group A and B patients.

Adverse events	Number	Percent
Visual disturbance	4	29
Photosensitivity	4	29
Fluorosis	2	14
Hallucination and photosensitivity	1	7
Hallucination	1	7
Weakness	1	7
Peripheral neuropathy	1	7
**Total**	**14**	**100**

### Posaconazole

Two patients were referred on posaconazole. During the 12 months, 26 patients (18 (14%) from Group A and 8 (13%) from Group B) were switched to posaconazole. All Group A and Group B patients who were changed to posaconazole stayed on therapy for the remainder of the 12 months of observation. One of the two patients who were referred while on posaconazole had to discontinue therapy due to isolation of a pan-azole resistant *A*. *fumigatus* isolate.

### Intravenous treatment

Eleven (8%) of the 132 group A patients received at least one course of intravenous (IV) therapy; three received IV micafungin and the remaining 8 received IV liposomal amphotericin B. Only one patient with adverse events to both voriconazole and itraconazole received 2 courses of IV micafungin, the rest received a single course of either IV micafungin or liposomal amphotericin B. Of the 3 patients who received IV micafungin, 1 had a pan-azole resistant *Aspergillus fumigatus* isolate, 1 had clinical failure on itraconazole and the third had adverse events to both itraconazole and voriconazole. The indications for IV liposomal amphotericin B were clinical failure on itraconazole (n = 2), multi-azole resistant isolate of *A*. *fumigatus* (n = 1) and intolerance to itraconazole (n = 5).

Five (8%) of the 64 Group B patients received intravenous antifungal therapy; three patients micafungin and two liposomal amphotericin B. Indications included pan-azole resistance (n = 2), 2, pan-azole drug intolerance (n = 2), and lack of clinical improvement due to failure to achieve therapeutic levels with all available azoles (n = 1).

### Surgery and bronchial artery embolization

Seven patients underwent surgical resection for treatment of CPA; two of these were among the ten patients who were not initiated on antifungal therapy (they had simple aspergilloma). Nine patients had bronchial artery embolization (BAE) done. Two of the 9 patients who had BAE also required surgery for uncontrolled haemoptysis.

### Outcomes

The treatment outcomes evaluated in this study includes change in quality of life scores (St George’s QoL questionnaire), MRC dyspnoea score, weight and *Aspergillus*-specific IgG at 6 and 12 months following initiation of therapy as summarised in Tables 4, 5 and 6.

**Table 4:** Weight, *Aspergillus* IgG, quality of life score, and MRC dyspnoea scores for all patients evaluated at baseline, 6 and 12 months. N = 206.

	Weight/kg	IgG/mg/L	SGRQ/100	MRC/5	Change from baseline weight	Change from Baseline IgG
	Median (range)	Mean (SD)	Median (range)	Median (range)	Mean (SD)	Mean (SD)
**Baseline**	62.2 (29.1–116.8)	259.7 (±295.0)	62.2 (2.9–98.2)	3 (1–5)	N/A	N/A
**6 months**	63.0 (34–119)	184.6 (±238.9)	60.0 (1.6–100)	3(1–5)	-0.34 (±3.6)	-60.0 (±190.7)
**12 months**	64.8 (32–108.4)	154 (±196.7)	57.2 (0.9–98.4)	3(1–5)	-0.33 (±5.2)	-92.8 (±204.2)

**Table 5:** Weight, *Aspergillus* IgG, quality of life score, and MRC dyspnoea scores for patients on any oral therapy at 12 months evaluated at baseline, 6 and 12 months. N = 140.

	Weight/kg	IgG/mg/L	SGRQ/100	MRC/5
	Median (range)	Mean (SD)	Median (range)	Median (range)
**Baseline**	63.0 (37.0–116.4)	265.1 (±286.7)	60.3 (2.9–97.5)	3(1–5)
**6 months**	64.5 (34–119)	202.2 (± 249.1)	56.9 (1.6–100)	3 (1–5)
**12 months**	65.8 (32.0–103.0)	158.1 (±192.6)	56.3 (0.9–95.1)	3 (1–5)

**Table 6:** Weight, *Aspergillus* IgG, quality of life score, and MRC dyspnoea scores for patients not on any therapy at 12 months evaluated at baseline, 6 and 12 months. N = 56.

	Weight/kg	IgG/mg/L	SGRQ/100	MRC/5
	Median (range)	Mean (SD)	Median (range)	Median (range)
**Baseline**	60.8 (29.1–116.8)	246.8 (±316.6)	67.5 (8.1–98.2)	3.5 (1–5)
**6 months**	58.9 (36–114)	144.0 (±210.2)	59.4 9.6–97.6)	3.0 (1–5)
**12 months**	61.0 (36.2–108.4)	145.8 (±207.2)	66.1 (16–98.4)	3.8 (1–5)

### Quality of life (SGRQ)

**Fig 2:**
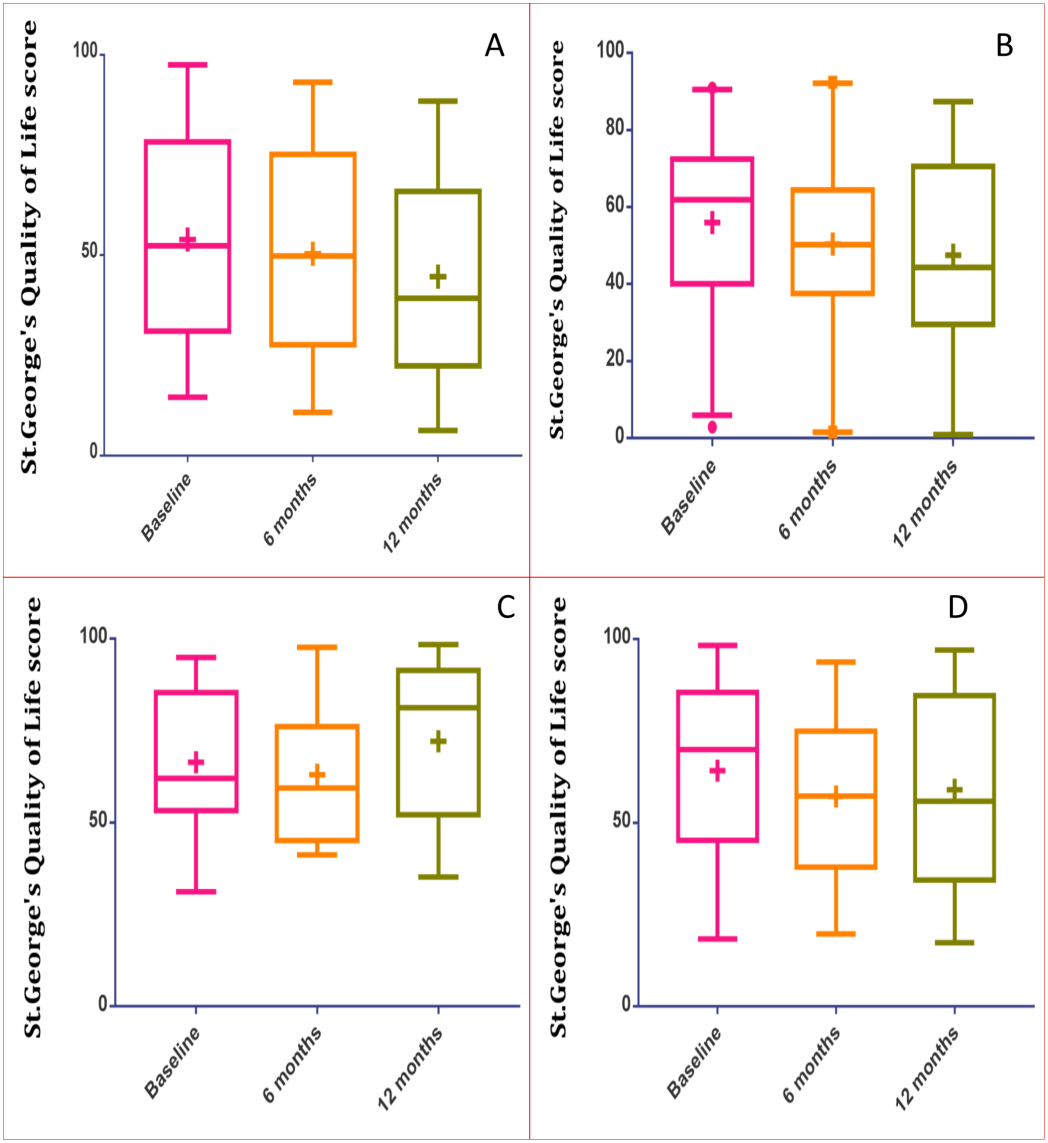
Change in St. George’s quality of life score at baseline, 6 months and 12 months for patients who were only on voriconazole (A) and itraconazole (B) for 12 months and for patients in whom voriconazole (C) or itraconazole (D) was discontinued. **Key**: **+**, mean score: **Middle bar**, Median.

The mean baseline SGRQ score for patients who continued on voriconazole for 12 months was 54.0 units (SD = 25.9) and this improved to 50.4 (SD = 26.7) and 44.7 (SD = 25.0) units at 6 and 12 months respectively while on treatment (Fig 2A). For patients who were on itraconazole for 12 months, the mean SGRQ score was 56.0 units (SD = 22.6) at baseline, 50.4 (SD = 23.5), and 47.6 units (SD = 25.8) at 6 and 12 months respectively (Fig 2B). For patients in whom voriconazole therapy was discontinued, the median SGRQ score was 66.4 (SD = 19.4), 63.0 (SD = 19.4), and 72.1 (SD = 22.4) units at baseline, 6 and 12 months (Fig 2C). Discontinuation of itraconazole produced median SGRQ scores of 64.2 (SD = 23.5), 57.2 (SD = 23.0) and 59.0 (SD = 26.8) at baseline, 6 and 12 months respectively (Fig 2D).

### Weight

Mean weights (Kg) at baseline, 6 and 12 months for patients on voriconazole were 71.6 (SD = 19.8), 71.3 (SD = 20.4), and 73.2 (SD = 14.2) Kg respectively. For patients who were on itraconazole this was 62.7 (SD = 15.9), 65.7 (SD = 15.4), 66.9 (SD = 13.2) respectively (S1 Table). For patients in whom therapy was discontinued, mean weights were 65.8 (SD = 8.1), 61.5 (SD = 6.4), and 63.0 (SD, 9.4) at baseline, 6 and 12 months respectively for those who discontinued voriconazole and 56.6 (SD = 11.0), 58.8 (SD = 9.2), 60.0 (SD = 9.7) at baseline, 6, and 12 months respectively for those who discontinued itraconazole therapy (S1 Table). Three outliers were noted in our dataset, 2 patients loss more than 10Kgs and one gained nearly 15kgs at 6 months and this was not the case at 12 months.

### MRC dyspnoea score

Breathlessness improved in those who stayed on therapy for 12 months, and in those who discontinued itraconazole. The median values of MRC dyspnoea score for patients who were on voriconazole for 12 months were 4, 3.5 and 3 units at baseline, 6 and, 12 months respectively and 3, 3 and 2 units at baseline, 6 and, 12 months respectively for patients who were on itraconazole for 12 months. The median values were 4, 4, and 5 units at baseline, 6 months and 12 months respectively for patients who discontinued voriconazole; this was 4, 3, and 3 units at baseline, 6 months and 12 months respectively for those in whom itraconazole discontinued (S2 Table).

### Serology

**Fig 3:**
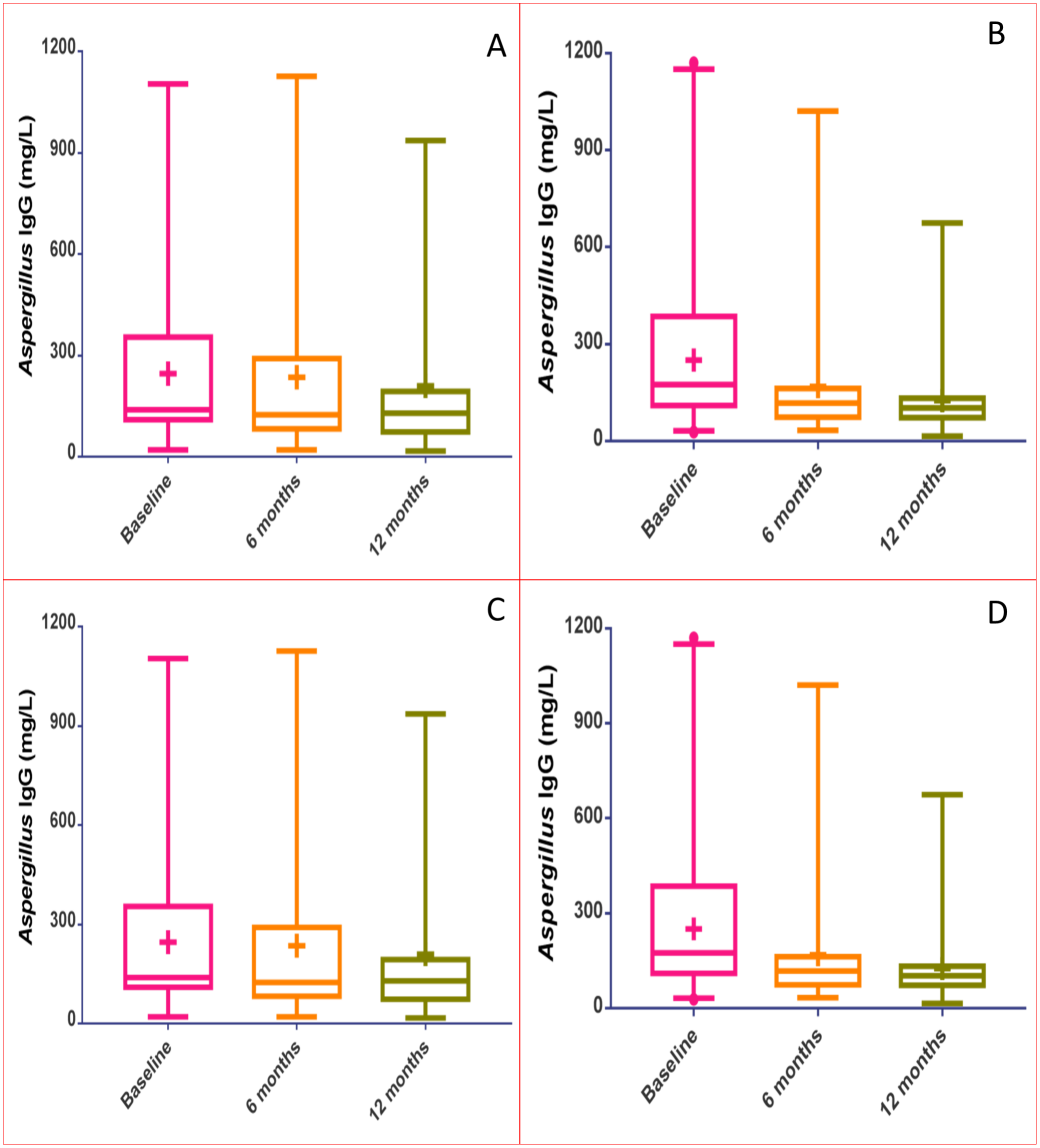
Variations in the *Aspergillus*-specific IgG at baseline, 6 months and 12 months for patients who were only on voriconazole (A) or itraconazole (B) for 12 months and for patients in whom voriconazole (C) or itraconazole (D) was discontinued. **Key**: **+**, mean score.: **Middle bar**, Median.

Mean serum *Aspergillus*-specific IgG levels (mg/L) decreased from 250.6 (SD = 239.6) at baseline to 168.8 (SD = 184.1) at 6 months, finally reaching 125.3 (SD = 119.4) mg/L at 12 months for patients who were on itraconazole for 12 months (Fig 3B). A corresponding decline in IgG levels was also observed for patients who continued on voriconazole for 12 months where the mean baseline IgG level decreased from 246.5 (SD = 251.8), to 235 (SD = 272.0) mg/L by 6 months and finally 210.1 (SD = 240.9) mg/L by 12 months (Fig 3A). Patients who had their itraconazole therapy discontinued had an initial decline in their mean serum IgG levels from 223 (SD = 238.5) at baseline to 131 (SD = 155.6) mg/L at 6 months and later a slight increase in serum IgG levels to 146.8 (SD = 212.4) mg/L by 12 months (Fig 3D). Contrarily, mean serum IgG for patients in whom voriconazole was discontinued consistently declined from 379.1 (SD = 533.0) at baseline to 275.9 (SD = 413.6) mg/L by 6 months, by 12 months it was 193.4 (SD = 262.6) mg/L (Fig 3C). The large SD observed could be explained by the over 1,000mg/L decline in IgG levels seen in 2 patients both at 6 months and 12 months.

### Emergence of resistance during therapy

In Group A, eight (8%) of the 105 patients who were on itraconazole developed azole resistant *A*. *fumigatus* isolates (5 itraconazole mono-resistance and 3 pan-azole resistance) and one (4%) of the 27 patients who was on voriconazole developed a pan-azole resistant isolate of *A*. *fumigatus*. For the Group B patients, five (11%) of the 46 patients who were on itraconazole developed resistance and one (6%) of the 16 patients on voriconazole developed resistant isolates of *A*. *fumigatus*. One of the two patients who were on posaconazole developed a pan-azole resistant isolate of *A*. *fumigatus*. There was no statistically significant difference in the rate of emergence of resistance between Group A and Group B, for either itraconazole (8% vs. 11%, p = 0.512) or voriconazole (4% vs. 6%, p = 0.701).

### Discontinuation of triazole antifungal agents

In Group A, 29 (22%) patients discontinued therapy. 6 were on voriconazole and 23 were on itraconazole. In Group B, 27 (42%) patients discontinued therapy, 21 of these patients were on itraconazole, 5 on voriconazole, and 1 on posaconazole. Azole discontinuation rates were higher in Group B than in Group A (p = 0.003).

### Survival

**Fig 4:**
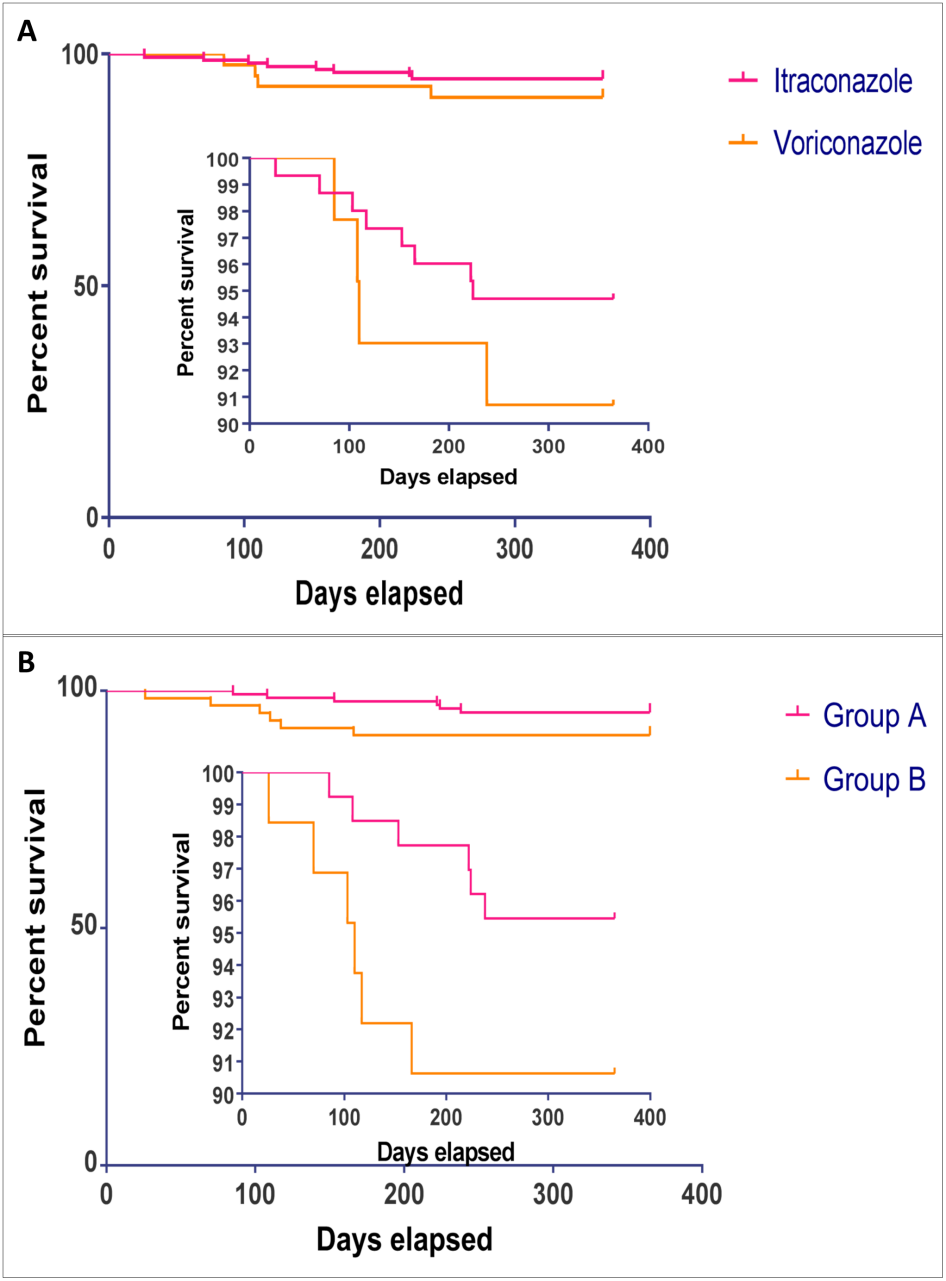
Kaplan-Meier survival plots for chronic pulmonary aspergillosis patients who were commenced on itraconazole and voriconazole as primary therapy (4A) and for Group A and B patients (4B). There was no statistical difference in mortality across primary therapy antifungal agents (log-rank, p = 0.462) and across the 2 groups (log-rank, p = 0.173).

Overall survival for all patients was 94% (194 of 206). Survival was 95% (140 of 148) in the itraconazole group and 92% (44 of 48) in the voriconazole group as shown in Fig 4A below (log-rank, p = 0.462). Across Groups A and B, survival was 95% (126 of 132) in Group A and 91% (58 of 64) in Group B as shown in Fig 4B below (log-rank, p = 0.173). The median duration of follow up between starting treatment and death was 15.6 (range 4–34) weeks, in those who died.

## Discussion

The present study shows that antifungal treatment for at least 12 months in CPA results in improvement in quality of life for patients initiated on voriconazole or itraconazole. This is in keeping with previous observations that long-term triazole therapy is the mainstay of management of CPA [12,13,16]. Adverse events are a frequent cause of discontinuation or change of therapy, and were seen in more than a third of cases.

The SGRQ score measures three main domains related to patient symptomatology, limitation in activities of daily living, and physical and psychological impact of airway disease, reflecting impaired health. It has been validated in a number of respiratory conditions including asthma, COPD, bronchiectasis [17,18] and recently CPA [19] with consistency and good repeatability. SGRQ score significantly correlates with MRC dyspnea score and disease severity in CPA [19]. In an asthma study, a mean score change of 4 units was associated with clinical response as assessed by patients, whereas in a COPD study, a change of 4.8 units was seen in patients who described the treatment as effective [20–22]. In this study, continuous treatment for 12 months with either itraconazole or voriconazole resulted in mean improvement of SGRQ scores of 8.4 units and 9.3 units respectively, whereas discontinuation or switching to another antifungal resulted in deterioration. These data provide further evidence of modest efficacy of azole treatment for CPA with respect to quality of life.

The MRC dyspnea scale is a frequently used measure of disability in cardio-respiratory diseases including CPA. However it is insensitive to change and has no definite/precise distinction between the grades [23]. In this audit, 17% of patients could not classify themselves accurately. There were small mean improvements in breathlessness overall, and in those on 12 months of azole therapy. CPA patients often have intercurrent bacterial infections [3] which have considerable negative effects on MRC scores and weight, usually temporarily. Other significant illnesses also can impact on quality of life and we have not explicitly analyzed these issues.

Weight gain is widely used as a clinical marker of response treatment in respiratory infection such as tuberculosis, and is also used as a surrogate marker in CPA. Interestingly, itraconazole use for 12 months was associated with weight gain (median difference in weight of 4.2 Kgs) while this was minimal (median difference in weight of 1.6 Kgs) with voriconazole use. This may reflect clinical improvement but could also be partly explained by peripheral fluid retention which is a common side effect of itraconazole. Some patients may have subtle toxicity with voriconazole, insufficient to stop them taking the drug, but impairing their appetite, as reported verbally in those who stopped therapy. Therefore, we have not been able to demonstrate that weight gain is associated with improvement in SGRQ scores.

An elevated level of *Aspergillus*-specific IgG is fundamental to the diagnosis of CPA [1]. The currently available tests for *Aspergillus*-specific IgG have wide variability with coefficient of variation (CV) between 3.4–43.7% [24]. The Immunocap^®^ assay has a coefficient of variation (CV) of 13%. Overall, the mean baseline IgG levels were well above (5.6–9.5 times) the upper reference limit (40mg/L). Patients started on voriconazole had a higher baseline IgG, perhaps reflecting higher initial burden of disease. At 12 months, a decline in the IgG levels was found in all patients. Stopping itraconazole therapy resulted in rising levels of IgG, whereas this effect was not observed in patients stopping voriconazole, in whom *Aspergillus*-specific IgG levels continued to decline following treatment cessation. There was no correlation between quality of life score and serology. At present, there is no widely accepted consensus on the use of serology to document response to therapy. Our results show that antifungal therapy is associated with a reduction in IgG levels, and stopping therapy may result in a rebound; whether this translates to clinical response or failure of treatment needs further study.

The best antifungal agent for initial treatment of CPA is not known. Itraconazole is often the preferred agent because of cost issues, followed by the newer triazoles. We observed similar improvement in scores in patients started on itraconazole or voriconazole; however, patients on voriconazole typically had a higher burden of disease radiologically and clinically (this is supported by the higher baseline *Aspergillus*-specific IgG level in patients on voriconazole). The reported efficacy of oral itraconazole is somewhat variable, with a range of 30–93%, duration of administration approximately 4–12 months, and adverse effects in 16–33% of patients [11,13,25,26]. The response rate in voriconazole therapy ranges from 13–65% with administration for several months, also with some adverse events in 6–21% notably visual disturbances, photosensitivity, hepatic toxicities and blurred vison [27–30]. Posaconazole is likely to be at least as effective [10,16]. We observed similarly high rates of discontinuation because of side effects (37% for voriconazole vs. 33% for itraconazole) indicating the need for better, less toxic agents. In our series, no patient had to discontinue posaconazole, although numbers were small.

Interestingly, 13% (n = 19) of the 151 patients who were on itraconazole stopped because of microbiological failure (resistance) compared to 5% (n = 2) of the 43 patients on voriconazole. It is possible that voriconazole may have a higher threshold for development of resistance. The presence of azole-resistant *Aspergillus spp*. isolates is becoming a major threat to CPA management. However, azole resistance is frequently under-recognized or inadequately diagnosed in CPA, partly because fungal culture is insensitive [31]. The development of resistance is a complication of prolonged azole treatment, and particularly associated with low serum concentrations of itraconazole and the presence of an aspergilloma [32–35]. Haemoptysis may be a sign of therapeutic failure and/or antifungal resistance [2]. Maximizing exposure to antifungal agents and minimizing adverse events is essential in the long-term management of CPA. Therapeutic drug monitoring (TDM) helps guide this [36]. Patients on long-term oral triazoles often require multiple dose changes tailored to TDM to optimize efficacy and minimize toxicity. In this study, around half of patients needed dose adjustments.

Sixteen (8%) of our patients were treated with either intravenous micafungin or liposomal amphotericin B therapy. With the increasing incidence of azole-resistant *A*. *fumigatus* isolates, clinical failure, intolerance and /or adverse events to first and second line agents used in the treatment of CPA, alternative management strategies for CPA such as short or long courses of IV liposomal amphotericin B and micafungin are considered [12]. About 74% of patients treated with short course IV liposomal amphotericin B have been reported to show clinical response [37]. With short course IV micafungin therapy, 2 studies reported short term clinical and radiological responses between 55% and 68.4% [38,39]. The clinical efficacy of intravenous micafungin and caspofungin for the treatment of CPA are similar [40]. There are no published data on anidulafungin for the treatment of CPA.

In conclusion, continuous treatment with itraconazole or voriconazole for at least 12 months resulted in the greatest benefit in terms of quality of life scores in CPA. *Aspergillus* Ig G also improved on treatment, outcomes were similar for both agents, although voriconazole may be preferred for patients with a higher burden of disease, for example multiple aspergillomas or higher *Aspergillus* serology. However azole toxicity is common and the drugs are poorly tolerated resulting in difficulties in continuing the lifelong therapy these individuals require in order to prevent ongoing lung damage and parenchymal fibrosis. Coupled with the threat of emerging azole resistance and the difficulties associated with defining treatment outcomes in a heterogeneous, multi morbid, CPA population, further prospective studies addressing the impact of azole therapy on CPA, and the identification of appropriate biomarkers with which to measure therapeutic response, are required to fully ascertain the impact of azole therapy on progression of disease.

## Supporting information

S1 Table Changes in weight at baseline, 6 months, and 12 months for all patients, patients who remained on the same therapy and for those in whom therapy was discontinued. (DOCX)

S2 Table Variations in the MRC dyspnea scores at baseline, 6 months, and 12 months for all patients, patients who remained on the same therapy and for those in whom therapy was discontinued. (DOCX)

S1 File Dataset. (XLSX)
