# Supplementary material for: Twelve-month clinical outcomes of 206 patients with chronic pulmonary aspergillosis
Source: PLoS One. 2018 Apr 10;13(4):e0193732. doi: 10.1371/journal.pone.0193732 (PMC5892866; doi:10.1371/journal.pone.0193732)
Supplement: S1 Table — (DOCX) [file pone.0193732.s001.docx]

**S1 Table**. Changes in weight at baseline, 6 months, and 12 months for all patients, patients who remained on the same therapy and for those in whom therapy was discontinued.

|  | Itraconazole  (All, n=148) | Voriconazole  (All, n=46) | Itraconazole  (same, n=56) | Voriconazole  (same, n=21) | Itraconazole  (discontinued, n=44 ) | Voriconazole  (discontinued, n=11) |
| --- | --- | --- | --- | --- | --- | --- |
|  | Median (range )/Kg | Median (range )/Kg | Median (range )/kg | Median (range )/Kg | Median (range )/kg | Median (range )/Kg |
| Baseline | 61.2 (29.1-116.8) | 68.5 (42.9-116.4) | 61.2 (39.0-114.8) | 68.5 (42.9-116.4) | 57.1 (29.1-77.8) | 67.2 (54.0-81.8) |
| 6 months | 61.6 (34.1-114.2) | 57.2 (41.1-119.4) | 65.8 (35.9 -111.0) | 70.2 (41.0-119.4) | 58.1 (43.0-80.2) | 61.8 (51.4-69.4) |
| 12 months | 61.2 (32.0-108.4) | 70.4 (44.4-103.0) | 66.4 (41.8 -99.2) | 74.4 (44.4-103.0) | 59.1 (37.0-80.6) | 65.4 (51.0-77.2) |
