# Supplementary material for: Twelve-month clinical outcomes of 206 patients with chronic pulmonary aspergillosis
Source: PLoS One. 2018 Apr 10;13(4):e0193732. doi: 10.1371/journal.pone.0193732 (PMC5892866; doi:10.1371/journal.pone.0193732)
Supplement: S2 Table — (DOCX) [file pone.0193732.s002.docx]

**S2 Table**. Variations in the MRC dyspnoea scores at baseline, 6 months, and 12 months for all patients, patients who remained on the same therapy and for those in whom therapy was discontinued

|  | Itraconazole  (All, n=148) | Voriconazole  (All, n=46) | Itraconazole  (same, n=56) | Voriconazole  (same, n=21) | Itraconazole  (discontinued, n=44 ) | Voriconazole  (discontinued, n=11) |
| --- | --- | --- | --- | --- | --- | --- |
|  | Median (range ) | Median (range ) | Median (range ) | Median (range ) | Median (range ) | Median (range ) |
| Baseline | 3 (1-5) | 4 (1-5) | 3 (1-5) | 4 (1-5) | 4 (1-5) | 4 (1-5) |
| 6 months | 3 (1-5) | 4 (1-5) | 3 (1-5) | 3.5 (1-5) | 3 (1-5) | 4 (1-5) |
| 12 months | 3 (1-5) | 4 (1-5) | 2 (1-5) | 3 (1-5) | 3(1-5) | 5 (1-5) |
